# Evaluation of CD4^+^ T Lymphocyte Counts to Predict Survival of ICU Patients with Sepsis Using Sepsis-3 Criteria: A Prospective Cohort Study

**DOI:** 10.1155/2024/4293700

**Published:** 2024-08-26

**Authors:** Guoge Huang, Xusheng Li, Chunmei Zhang, Haizhong Li, Mengling Jian, Chunyang Huang, Yingqin Zhang, Luhua Xian, Hongke Zeng, Yuanyuan Xia, Wenqiang Jiang

**Affiliations:** ^1^ Guangdong Cardiovascular Institute Emergency Intensive Care Unit Department of Emergency Medicine Guangdong Provincial People's Hospital (Guangdong Academy of Medical Sciences), 106 Zhongshan Er Road, Guangzhou 510080, Guangdong, China; ^2^ Emergency Intensive Care Unit Department of Emergency Medicine Guangdong Provincial People's Hospital (Guangdong Academy of Medical Sciences), 106 Zhongshan Er Road, Guangzhou 510080, Guangdong, China; ^3^ Department of Emergency Boai Hospital of Zhongshan, No. 6 Chenggui Road, Zhongshan 528403, Guangdong, China; ^4^ School of Medicine South China University of Technology Guangzhou University City, Guangzhou 510006, Guangdong, China; ^5^ Department of Laboratory Medicine Guangdong Provincial People's Hospital Academy of Medical Sciences, Guangzhou, China; ^6^ Medical Oncology Department The Fifth Affiliated Hospital of Guangzhou Medical University Guangzhou Medical University, Guangzhou 510700, China

## Abstract

**Background:**

Sepsis remains a major health condition with a high mortality rate that may be related to immunosuppression. T lymphocyte subsets may reflect the immune function of sepsis patients. The purpose of this study was to investigate the predictive value of CD4^+^ T lymphocyte counts of ICU patients for their short-term prognosis.

**Methods:**

We conducted a prospective, observational cohort study in a general ICU and enrolled patients with sepsis using the Sepsis-3 criteria. Peripheral blood samples were collected within 24 hours of enrollment or measurement of blood cell analysis and biomarkers of CD4^+^ T lymphocytes and CD8^+^ T lymphocytes. Severity was classified by the Acute Physiology and Chronic Health Evaluation II and Sequential Organ Failure Assessment (SOFA) scores. The primary outcome was 28-day mortality.

**Results:**

A total of 100 patients with sepsis were enrolled and analyzed. CD4^+^ T lymphocyte counts gradually decreased based on 28-day mortality (*p* < 0.001). Similarly, multivariate logistic regression analysis showed that only CD4^+^ T lymphocyte counts were an independent predictor of 28-day mortality in sepsis patients. The area under the receiver operating characteristic curve of the combination of CD4^+^ T lymphocyte counts and the SOFA score was 0.78.

**Conclusion:**

Our study demonstrated that CD4^+^ T lymphocyte counts are associated with 28-day mortality. A combination of CD4^+^ T lymphocyte counts with the SOFA score increased the predictive accuracy for 28-day mortality.

## 1. Background

Sepsis is presently defined as a life-threatening organ dysfunction syndrome instigated by a dysregulated host response to infection, a condition that is garnering escalating interest in the field of emergency medicine [1]. Across the globe, sepsis is now estimated to result in more than 11 million deaths a year, and septic shock, the most severe form, leaves nearly 40% of patients dead at hospital discharge [2]. Over the past few decades, the mortality rate of sepsis has seen a gradual decrease due to the timely administration of antibiotics, fluid resuscitation, and multiple organ support therapies [3]. However, mortality rates persistently remain high among specific populations, such as immunosuppressed individuals. The Sequential Organ Failure Assessment (SOFA) score often falls short in identifying these patients, leading to a large segment of sepsis patients being erroneously classified as “immunocompetent” [4]. This misclassification results in prolonged intensive care unit (ICU) stays and increased financial burdens for these immunosuppressed patients.

To better identify these at-risk populations, numerous new technologies have been developed [5]. As an important immune cell, CD4^+^ T cells may play a significant role during sepsis. CD4^+^ T cells are a subgroup of lymphocytes that coordinates the cells of the immune system by releasing cytokines that are essential for their proliferation, differentiation, and bactericidal functions against pathogens [6]. It remains contentious whether CD4^+^ T cells are intimately involved in the early stages of sepsis. Some animal studies have shown that CD4^+^ T cells may directly mediate the host response to sepsis [6, 7], while others have shown that they had no impact on the inflammatory response [8]. Human patients and experimental mouse models of sepsis exhibit profound CD4^+^ T-cell apoptosis [9, 10]. However, the clinical relevance of these findings has not been sufficiently corroborated by research studies. Therefore, evaluating the relationship between sepsis and T lymphocyte subsets not only can it help to better understand the pathogenesis of sepsis-related immunity but it can also provide new insights as well as strategies for the treatment of sepsis. The purpose of this study was to investigate the predictive value of CD4^+^ T lymphocyte counts in peripheral blood using the Sepsis-3 criteria on 28-day mortality.

## 2. Methods

### 2.1. Patients

The study was performed in the general intensive care unit (ICU) of a 2,700-bed tertiary-level general hospital. From June 2016 to September 2022, we prospectively enrolled patients with sepsis. Patients admitted to the ICU were screened for inclusion and exclusion criteria and enrolled on ICU day 1 or 2. The inclusion criteria were as follows: patients (a) met Sepsis-3 criteria and (b) aged 18 years or older than 18 years. The exclusion criteria were as follows: (a) terminal stage of chronic diseases; (b) patients with autoimmune disease, immunodeficiency, or long-term use of immune suppressants; (c) patients with tumor; and (d) consent unable to be obtained. This study was approved by Guangdong Provincial People's Hospital Ethics Committee. Written informed consent was obtained from the patients or their immediate family. Consents for patients who were unable to provide consent were provided by their immediate relatives.

### 2.2. Data Collection

Clinical characteristics of patients, including age, gender, preexisting clinical conditions, vital signs, and results of laboratory examinations, were recorded on study days 1–7. SOFA and Acute Physiology and Chronic Health Evaluation II (APACHE II) scores were calculated. Patients were followed for at least 28 days after recruitment or until death. According to the mortality within 28 days, the patients were divided into nonsurvival and survival groups.

Blood samples were obtained in the morning within 24 hours after being enrolled. Study day 1 was defined as the day when the patients were enrolled. All patients underwent blood sampling on study day 1, and some of them who agreed also had their blood samples taken on study days 2, 4, and 7.

### 2.3. Flow Cytometry

Peripheral blood was extracted from all the patients and was collected in heparin tubes. The samples were transported to the laboratory at 4°C within 4 h. Erythrocytes were lysed, and cells were processed for evaluation by a researcher who was blinded to our clinical data. Antibodies were purchased from BD Pharmingen (Franklin Lakes, NJ). The manufacturer's instructions with respect to the use of monoclonal antibodies and their fluorescence minus one controls were followed: aliquots of 100 *μ*L of whole blood were incubated with PC-5.5-labeled anti-CD3 (2 *μ*L clone UCHT1), FITC-labeled anti-CD4 (2 *μ*L clone RPA-T4), and PE-labeled anti-CD8 (2 *μ*L clone HIT8a). Samples were processed on CytoFLEX (Beckman Coulter, Inc.) and analyzed using CytExpert Software version 2.0 (Beckman Coulter, Inc.). Lymphocytes were gated by forward scatter (FSC) and side scatter (SSC), and T-cell subsets were further identified by CD3^+^, CD4^+^, and CD8^+^ staining. Results are expressed as percentages.

### 2.4. Statistical Analysis

We used statistical software SPSS for Windows version 13.0 (SPSS Inc., Chicago, IL, USA) for all statistical analyses. The baseline characteristics were described as frequencies, percentages, median, and interquartile ranges. Comparisons between groups were made using the Pearson *χ*^2^ test for categorical data and the nonparametric Mann–Whitney *U* test for continuous variables. Repeated measurement data analysis of variance was performed to show the dynamic changes in CD4^+^ T lymphocyte counts. Binary logistic regression was used to identify the variables associated with 28-day mortality in patients with sepsis. Those variables with a collinear relationship were not included in the multivariable analysis. CD4^+^ T lymphocyte counts were stratified using the optimal threshold indicated by the receiver operating characteristic (ROC) curve. Patient survival was analyzed using the Cox proportional hazards model. All statistical tests were two-tailed, and *p* < 0.05 was considered statistically significant.

## 3. Results

### 3.1. Patient Characteristics

**Figure 1:**
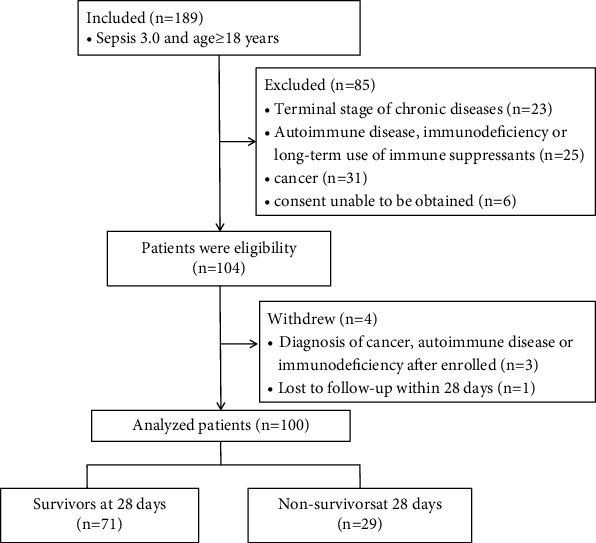
Study profile.

The study enrolled 104 patients, and 4 patients were withdrawn because 3 of them were eventually diagnosed with cancer or autoimmune diseases and 1 lost to follow-up (Figure 1). Thus, 100 patients were analyzed. There were 72 men (72%) and 28 women (28%), all of whom were Chinese, and the median age was 72 (60–78) years. A total of 29 patients died at or before 28 days. The demographic and clinical characteristics are shown in Table 1.

### 3.2. Comparison of Blood Cell Analysis, N/L, and Lymphocyte Subsets of Survivors and Nonsurvivors in Patients with Sepsis

**Figure 2:**
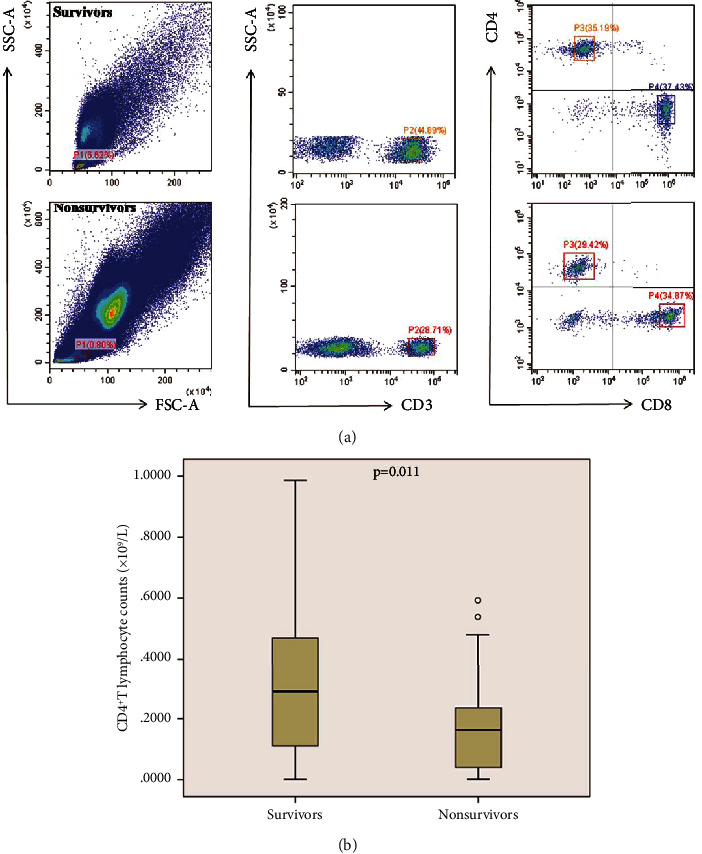
Comparison of CD4^+^ T lymphocyte counts in survivors and nonsurvivors according to 28-day mortality. (a) Representative flow dot plots of the lymphocyte gating strategy and the percentage of CD4^+^ T lymphocyte and CD8^+^ T lymphocyte in survivor and nonsurvivor groups. (b) Box-plot representation of CD4^+^ T lymphocyte counts (×10^9^/L). Data are shown as box plot with medians (lines inside boxes) and 25th and 75th quartiles (limits of boxes); whiskers indicate the range. CD4^+^ T lymphocyte counts were significantly increased in survivors (*n* = 71) when compared with nonsurvivors (*n* = 29). CD4^+^ T lymphocyte counts = lymphocyte counts × P2 × P3; CD4^+^ T lymphocyte counts = lymphocyte counts × P2 × P4.

Patients were divided into survivors and nonsurvivors at 28 days. We compared the two groups of the patients, and the results are shown in Table 2. In nonsurvivors, CD4^+^ T lymphocyte counts were significantly lower than those in survivors (Table 2, *p* < 0.05; Figures 2(a) and 2(b)). Similar results were also observed when compared with the lymphocyte counts, CD8^+^ T lymphocyte counts, and lactate (Table 2; *p* < 0.05). The representative flow dot plots are shown in Figure 2(a).

### 3.3. Dynamic Changes in CD4^+^ T Lymphocyte Counts

**Figure 3:**
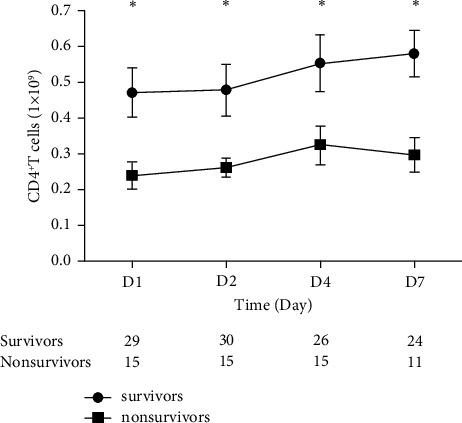
Time-serial measurements (mean ± SEM) of CD4^+^ T lymphocyte counts in survivors (square) and nonsurvivors (rhombus). Repeated measurement data analysis of variance was performed to show that CD4^+^ T lymphocyte counts in nonsurvivors were significantly lower than those in survivors (*p* < 0.05). Sphericity test of Mauchly (*W* = 0.92 and *p*=0.769). There was no significant difference between repeated factors at 4 different time points (*p*=0.163). Time had no bearing on the level of CD4^+^ T lymphocyte counts (*p*=0.901).

CD4^+^ T lymphocyte counts of 45 patients were measured on days 1, 2, 4, and 7 after being enrolled. CD4^+^ T lymphocyte counts in nonsurvivors were significantly lower than those in the survivors on every time point (Figure 3). CD4^+^ T lymphocyte counts continued to increase in the survivors, notably in the early disease course (1-4d). However, CD4^+^ T lymphocyte counts began to decline at 4 days in the nonsurvivors.

### 3.4. CD4^+^ T Lymphocyte Counts as an Independent Predictor of 28-Day Mortality in Sepsis Patients

Multivariate logistic regression analysis was used to identify the independent predictor of 28-day mortality. These parameters showed significant differences by the univariate analysis, but excluding the possible collinearity indicators between survivors and nonsurvivors, shock, heart rate, Lac, CD4^+^ T lymphocyte counts, and CD8^+^ T lymphocyte counts were included in this analysis. The results showed that CD4^+^ T lymphocyte counts, shock, and heart rate were independently associated with 28-day mortality. The detailed data are presented in Table 3.

### 3.5. ROC of CD4^+^ T Lymphocyte Counts for Predicting 28-Day Morality and Survival Analysis in Sepsis Patients

**Figure 4:**
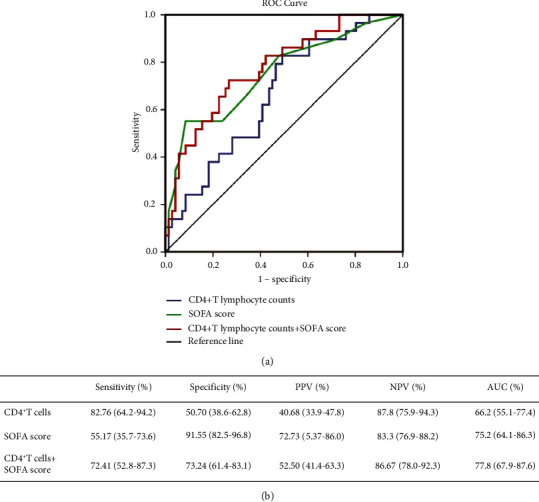
(a) Receiver operating characteristic (ROC) curve for predicting 28-day mortality in sepsis patients. (b) Comparison of sensitivity, specificity, positive predictive values (PPV), negative predictive values (NPV), and the area under the curve (AUC) of various parameters for predicting 28-day mortality.

The ROC curve obtained with CD4^+^ T lymphocyte counts has an AUC of 0.662 (CI 0.551–0.774), and the ROC curve obtained with the SOFA score has an AUC of 0.752 (CI 0.641–0.864). The combination of these two parameters has an AUC of 0.778 (CI 0.679–0.876) for predicting 28-day mortality (Figure 4(a)). ROC curve analysis showed that 0.2810 (×10^9^/L) of CD4^+^ T lymphocyte counts was the optimal threshold for predicting 28-day mortality in patients with sepsis. The sensitivity, specificity, positive and negative predictive values, and its corresponding confidence interval of 95% for the three ROC curves were used to estimate the prognostic accuracy. The detailed results are shown in Figure 4(b).

**Figure 5:**
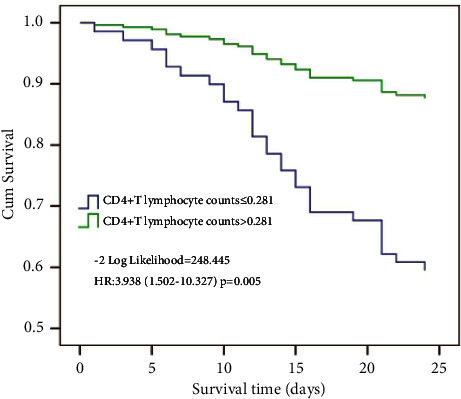
Survival curves of patients with sepsis stratified by CD4^+^ T lymphocyte counts. Survival curves showed that sepsis patients with CD4^+^ T lymphocyte counts not above 0.281 (×10^9^/L) had a lower probability of survival at 28 days (−2 log likelihood = 248.445; HR: 3.938 (1.502–10.327); *p*=0.005) compared to patients with higher levels.

Using cutoff values determined by the ROC curve, patients with the CD4^+^ T lymphocyte counts lower than 0.281 (×10^9^/L) had a lower probability of survival at day 28 than patients with higher CD4^+^ T lymphocyte counts (Figure 5).

## 4. Discussion

Previous studies found that the decrease in peripheral blood lymphocytes in sepsis patients is associated with the severity of the disease and the poor prognosis [11]. Martignoni found that CD4^+^ T cells may facilitate the early clearance of bacteria by regulating neutrophil function. The decrease of CD4^+^ T cells indicates the immunosuppression or immune paralysis of the organism [12]. Recently, the criteria for sepsis were updated [2] and the definition of sepsis was changed to a life-threatening organ dysfunction due to a dysregulated host response to infection (Sepsis-3). Changing the criteria may ultimately also affect the performance of prognostic biomarkers. As far as we know, the current study is one of the earliest studies correlating CD4^+^ T lymphocyte counts to a prognostic value of sepsis patients using the Sepsis-3 criterion.

In the present study, T-cell subgroups were selected as the target to explore their predictive performance on short-term mortality under the new sepsis criteria. We show here that CD4^+^ T lymphocyte counts and CD8^+^ T lymphocyte counts were significantly lower in nonsurvivors compared with nonsurvivors. Multivariate logistic regression analysis showed that only CD4^+^ T lymphocyte counts were independently associated with 28-day mortality in patients. Area under the curve of CD4^+^ T lymphocyte counts (0.662 and 0.551–0.774) indicated that it is an effective marker for predicting the short-term mortality of sepsis, although its predictive value was not too high.

Many biomarkers showed a relevant correlation with the clinical outcome of patients with sepsis, but their time courses may be more reliable than absolute levels [13]. Clinical studies had found that the immune status of sepsis was constantly changing, which is different from the early stage and the progression of diseases [14]. The present results have shown the dynamics change of CD4^+^ T lymphocytes on study days 1, 2, 4, and 7 of some patients with sepsis. Interestingly, the results showed that it is significantly associated with an increase in overall and sepsis-attributable mortality rates. As shown in time-serial measurements, CD4^+^ T lymphocyte counts in survivors were progressively increased. However, in the nonsurvivor group, the counts were increased at the beginning but decreased thereafter. This suggests that with the progression of the disease, the continued decline in CD4^+^ T lymphocyte counts is indicative of a poor prognosis of sepsis patients.

It is suggested that organ failure should be the core of sepsis. In this connection, SOFA is now widely accepted and is a relatively accurate measure of the severity of the disease [2]. As one of the scoring systems for sepsis patients, the SOFA score was proposed by scholars of ESICM (European Society of Intensive Care Medicine) in Paris in 1994, which has a history of 23 years [15]. Recently, Francesca's study reported that the SOFA score shows a moderate prognostic stratification ability in sepsis patients [16]. Our study showed a similar result that the area under the curve of the SOFA score for predicting 28-day mortality is 0.752 (0.641–0.863). In comparative analysis of sensitivity and specificity of CD4^+^ T lymphocyte counts versus SOFA score, we found that the former is more sensitive at 82.76% for the prognosis, while the latter is more specific at 91.55%. More importantly, we have demonstrated that the SOFA score when combined with CD4^+^ T lymphocyte counts was a better factor for predicting 28-day mortality compared with the SOFA score and CD4^+^ T lymphocyte counts alone. There are six scoring systems (respiration, coagulation, liver, cardiovascular, central nervous system, and renal) in the assessment of SOFA [14], but the systemic nature and inflammatory response of sepsis involved large number of organs and cell systems [17], which is far more than the 6 items included in the SOFA score. It has been proposed that future iterations of the sepsis definitions should include an updated SOFA score with more optimal variable selection, cutoff values, and weighting, or more superior scoring systems [2]. More and more evidence showed that the function of the immune system played an important role in the occurrence and development of sepsis, and it is closely related to the prognosis of patients [9, 18]. Thus, it can be confidently assumed that the immune system could and should be a part of the improvement of the SOFA score, and CD4^+^ T lymphocyte counts can be used as an indicator of assessment.

We have further shown that besides CD4^+^ T lymphocyte counts, shock and heart rate were also the independent risks associated with 28-day mortality. Consistent with our findings, a retrospective study by Hayase et al. reported that tachycardia was a significant and independent predictor of a reduced survival rate in sepsis patients [6]. Recent studies have shown that controlling heart rate can reduce mortality in patients with septic shock and have a significant effect [18]. Furthermore, Jayaprakash et al. found that elevated modified shock index during early sepsis is associated with the development of the SOFA score and mortality [18]. This indicated that cardiac function plays an important role in sepsis, underscoring therefore the needs to obtain the necessary information.

Sepsis is a heterogeneous biological syndrome which is incompletely understood. Many of the established and emerging biomarkers are helpful for sepsis patients [18]. Notwithstanding, we have identified a biomarker that can reflect the immune function, predict the prognosis of sepsis patients, and increase the predictive accuracy of the SOFA score. Mounting data show that, in most ICU patients, sepsis-associated immunosuppression is associated with increased morbidity and mortality [18]. The immunomodulatory therapy of sepsis is one of the focuses of the current research study. Taken together with previous studies [7, 11, 18], we believed that CD4^+^ T lymphocyte counts can also play an important role in monitoring the therapeutic effect of immune regulation.

However, there were several limitations in this study. Firstly, it was a single-center study and the sample size was relatively small. Secondly, our study enrolled only ICU patients with the diagnosis of sepsis and who had higher acuity than the general population of infectious patients. Thirdly, we did not carry out further research on the cellular function of the remaining CD4^+^ T lymphocytes. Finally, this study was lacking a healthy control group.

## 5. Conclusions

In conclusion, our study strongly supports that CD4^+^ T lymphocyte counts are associated with mortality in sepsis patients. CD4^+^ T lymphocyte counts in combination with the SOFA score significantly increase the predictive accuracy for 28-day mortality. Furthermore, measurement of CD4^+^ T lymphocyte counts is a promising independent prognostic marker for sepsis patients.

## Figures and Tables

**Table 1:** Baseline characteristics of the patients.

Parameters	All patients, *n* = 100	Nonsurvivors, *n* = 29	Survivors, *n* = 71	*p* value
*Demographic characteristics*				
Female, *n* (%)	28 (28)	8 (27.6)	20 (28.2)	0.953
Age (years)	72.00 (60.00–78.00)	72.00 (61.00–77.00)	72.00 (60.00–79.00)	0.918

*Laboratory findings*				
WBC (×10^9^/L)	14.97 (9.30–20.10)	11.86 (8.15–17.92)	15.94 (8.71–20.47)	0.118
Lac (mmol/L)	1.40 (1.10–2.00)	1.77 (1.23–2.56)	1.30 (1.10–1.80)	0.039
Bacteriology positive, *n* (%)	53 (53)	18 (62.1)	35 (49.3)	0.246

*Vital signs*				
T (°C)	37.15 (36.70–38.2)	37.40 (36.65–38.10)	37.00 (36.70–38.40)	0.985
Heart rate (cpm)	100.00 (85.25–110.00)	105.00 (97.50–110.00)	96.00 (82.00–110.00)	0.047
MAP (mmHg)	89.00 (80.00–99.33)	85.00 (79.00–98.17)	89.00 (80.33–100.00)	0.569

*Source of infection, n (%)*				
Bloodstream	4 (4)	1 (1.4)	3 (10.3)	0.072
Lungs	64 (64)	47 (66.2)	17 (58.6)	0.474
Abdomen	14 (14)	9 (12.7)	5 (17.2)	0.540
Urinary system	10 (10)	7 (9.9)	3 (10.3)	1.000
Central nervous system	4 (4)	4 (5.6)	0 (0)	0.320
Others	4 (4)	3 (4.2)	1 (3.4)	1.000

*Severity of illness*				
GCS score	12.00 (7.13–15.00)	8.00 (3.50–13.50)	12.00 (9.00–15.00)	<0.01
APACHE II score	15.00 (10.00–20.00)	19.00 (15.00–24.50)	13.00 (9.00–17.00)	<0.01
SOFA score	5.00 (4.00–8.00)	9.00 (5.00–12.00)	4.00 (3.00–6.00)	<0.01
Shock, *n* (%)	24 (24)	12 (41.4)	12 (16.9)	<0.01

Data are shown as median and interquartile range unless otherwise indicated. Pearson chi-square test was performed for sex, bacteriology positive, and shock while the nonparametric test of two independent samples (Mann–Whitney) were for the others. WBC: white blood cell, Lac: lactate, GCS: Glasgow Coma Scale, APACHE II: Acute Physiology and Chronic Health Evaluation, SOFA: Sequential Organ Failure Assessment, T: temperature, and MAP: mean arterial pressure.

**Table 2:** Comparison of blood cell analysis, N/L, and lymphocyte subsets of survivor and nonsurvivors groups.

Parameters	All patients	Nonsurvivors	Survivors	*p* value (N/S)
Number	100	29	71	
N (×10^9^/L)	13.01 (8.00–17.67)	10.55 (6.88–16.77)	13.58 (8.62–18.24)	0.26
L (×10^9^/L)	0.85 (0.48–1.30)	0.50 (0.37–1.07)	0.91 (0.62–1.40)	<0.01
N/L	13.04 (8.62–27.99)	14.01 (9.39–31.02)	11.82 (8.29–26.25)	0.248
CD4^+^ T cells counts (×10^9^/L)	0.19 (0.09–0.42)	0.16 (0.03–0.24)	0.29 (0.11–0.47)	0.011
CD8^+^ T-cell counts (×10^9^/L)	0.15 (0.06–0.30)	0.11 (0.02–0.25)	0.17 (0.06–0.30)	0.031
CD4/CD8	1.65 (0.96–2.49)	1.66 (0.78–2.24)	1.63 (1.02–2.67)	0.144

Data are shown as median and interquartile range. Nonparametric test of two independent samples (Mann–Whitney) was performed for the comparison of nonsurvivors and survivors. N, neutrophils; L, lymphocyte; N/L, the ratio of neutrophil count to lymphocyte count.

**Table 3:** Logistic regression analysis of independent risk factors for 28-day mortality.

Variable	*B*	SE	Wald	*p* value	Odds ratio	95% confidence interval for *B*	
						Lower limit	Upper limit
CD4^+^ T cells	−2.726	1.333	4.181	0.041	0.066	0.005	0.893
Heart rate	0.039	0.015	6.692	0.010	1.040	1.010	1.072
Shock	1.157	0.591	4.104	0.043	3.181	1.038	9.747
Constant	−4.565	1.605	8.093	0.004	0.010		

## Data Availability

The data that support the findings of this study are available from the corresponding author upon reasonable request.

## Abbreviations

APACHE II: Acute Physiology and Chronic Health Evaluation II
CI: Confidence interval
GCS: Glasgow Coma Scale
ICU: Intensive care unit
Lac: Lactate
MAP: Mean arterial pressure
PBMCs: Peripheral blood mononuclear cells
SOFA: Sequential Organ Failure Assessment
T: Temperature
WBC: White blood cell.
